# The effectiveness of formative assessment for enhancing reading achievement in K-12 classrooms: A meta-analysis

**DOI:** 10.3389/fpsyg.2022.990196

**Published:** 2022-08-22

**Authors:** Qianying Xuan, Alan Cheung, Dan Sun

**Affiliations:** The Chinese University of Hong Kong, Hong Kong, Hong Kong SAR, China

**Keywords:** reading achievement, K-12 students, differentiated instruction, meta-analysis, formative assessment

## Abstract

This quantitative synthesis included 48 qualified studies with a total sample of 116,051 K-12 students. Aligned with previous meta-analyses, the findings suggested that formative assessment generally had a positive though modest effect (ES = + 0.19) on students’ reading achievement. Meta-regression results revealed that: (a) studies with 250 or less students yielded significantly larger effect size than large sample studies, (b) the effects of formative assessment embedded with differentiated instruction equated to an increase of 0.13 SD in the reading achievement score, (c) integration of teacher and student directed assessment was more effective than assessments initiated by teachers. Our subgroup analysis data indicated that the effect sizes of formative assessment intervention on reading were significantly different between Confucian-heritage culture and Anglophone culture and had divergent effective features. The result cautions against the generalization of formative assessment across different cultures without adaptation. We suggest that effect sizes could be calculated and intervention features be investigated in various cultural settings for practitioners and policymakers to implement tailored formative assessment.

## Introduction

In an era of reconfiguring the relationship between learning and assessment, spurred by quantitative and qualitative evidence, formative assessment is proffered to meet the goals of lifelong learning and promote high-performance and high equity for all students (OECD, 2008). It has gained momentum among researchers and practitioners in various culture contexts. In an oft-cited ‘configurative review’ (Sandelowski et al., 2012) on formative assessment, Black and Wiliam (1998) reported that effect sizes of formative assessment of student achievements were between 0.4 and 0.7 ranging over age groups from 5-year-olds to university undergraduates. The impact of teachers’ formative evaluation on student achievement was ranked third with an effect size of 0.9 in 138 learning activities influencing student achievement (Hattie, 2009). Also, feedback, as an essential part of formative assessment, has been found to positively enhance students’ learning (Hattie and Timperley, 2007; Hattie, 2009; Wisniewski et al., 2019). The large *prima facie* effect sizes found to raise the standards of learning laid a foundation for future evidence-based assessment policy reform. Formative assessment has gained an ever-widening array of attentions in various countries and regions.

In the past three decades, only four comprehensive reviews have reported the positive effect sizes of formative assessment on reading achievement which ranged from +0.22 to +0.7 (Fuchs and Fuchs, 1986; Black and Wiliam, 1998; Kingston and Nash, 2011; Klute et al., 2017). Yet in a literature review of 15 studies commissioned by the Australian Institute of Teaching and School Leadership (AITSL), the researchers stated that the impact of formative assessment on reading achievement was discouraging due to no effective tools could be identified and some programs integrated with technologies (Lane et al., 2019). The interpretations from the prior meta-analyses and literature review seems to be conflicting. Different school subjects require domain-specific effective formative assessment interventions (Wiliam, 2011). Arguably, whether how formative assessment enhances students’ reading achievement remains unclear, this problem can be addressed by an updated and comprehensive meta-analysis. Lane et al. (2019) concerned that it could not distinguish the effect of formative assessment from digital technology on reading if they were mixed in a program. This issue can be settled by setting the involvement of digital technology in formative assessment practices as a moderator, which compares the programs with or without technology. Given the importance of formative assessment and the need for further statistical evidence on the reading subject (Clark, 2010; Van der Kleij et al., 2017; Black and Wiliam, 2018; Andrade et al., 2019), the purpose of this review, included literature in English and Chinese up to 2021, is to assess evidence from rigorous evaluations to determine the magnitude of experiment effects of formative assessment on students’ reading performance and identify what features influenced its effectiveness. Noticeably, performing international comparison of formative assessment practices requires culture sensitivity (Shimojima and Arimoto, 2017). In this meta-analysis, we set three factors suggested by Cheung et al. (2021) to frame the features of formative assessment: substantive factors (student characteristics, grade level, type of intervention, digital technology, program duration, differentiated instruction), methodological factors (sample size, research design), and other factors (publication type, cultural setting).

### Working definition of formative assessment

Since the term formative assessment has been used widely and diversely in the literature and because its classroom practice can vary within different educational settings, it is important to provide a working definition of the term to guide this review. Given the nebulous nature of formative assessment, a working definition of formative assessment is proposed based on the prior definitions in the past three decades. The essential statements of the 19 definitions (shown in Supplementary material) were compiled aligned with a succinct framework. Jönsson (2020) suggested that definition of formative assessment should include evaluative judgment (qualitative judgment) occurring in daily teacher-student interactions and a psychometric understanding of assessment depending on aggregating evidence of student learning collected by teachers. To follow this advice and identify potential studies, this review culls the more comprehensive descriptions under each element of the suggested definitions. Formative assessment in this review is broadly defined as

*an active and intentional process with formal and informal classroom practices/activities harvesting evidence of students’ learning progress by evaluative/qualitative judgment and a psychometric understanding of various assessments (what) during teaching and learning (when), in which teachers (who) continuously and systematically elicit, interpret and use evidence about students’ learning and conceptual organization (how) to guide their pedagogical plans (why), and/or students (who) work with/without teachers or peers to adjust their current learning tactics (how) with an effort to improve their achievements and self-regulate their learning (why)* (Popham, 2008; Black and Wiliam, 2009; Chappius, 2009; Moss and Brookhart, 2009; Cizek et al., 2019).

The evaluative judgment refers to the daily teacher- student interactions eliciting evidence about learners’ progress, for instance, feedback, discussions, presentations, and other students’ artifacts. Psychometric assessments entail some quizzes, tests or indirect measurement that necessitates interpretation of outcomes (Jönsson, 2020). In this sense, some formative utilities of benchmark assessments and summative assessments (Wiliam, 2011) would be included if they met all the selection criteria in this review. Considering that formative assessments are classroom practices to identify students’ learning gaps and improve their learning, participants can be teachers, students or their peers, as well as the integration of teachers and students. This review would clarify types of intervention to compare the effectiveness of different participants’ engagement.

Alternative terminologies have emanated from different emphases to serve a common underlying formative purpose (Kingston and Nash, 2011). It is worth mentioning, the term assessment for learning (AfL) is often used interchangeably with formative assessment to emphasize the function of formative assessment to improve student learning (Heritage, 2010; Bennett, 2011). The term AfL, first used by Harry Black (Black and Wiliam, 1986), was advocated by the Assessment Reform Group (ARG) in United Kingdom. Another term assessment as learning (AaL), was also phrased to signal the active role students play in the formative assessment process (Earl, 2012). Regarding assessment for, as, and of learning, each delineates the purpose for which the assessment was carried out. Differently, formative assessment and summative assessment are clarified by the functions they actually serve (Wiliam, 2011). Bennett (2011) suggested that it was not instructive to equate AfL with formative assessment and assessment of learning with summative assessment. However, a thorough exploration of the nuances between these two distinctions is beyond the scope of this paper. To include potentially qualified studies as broadly as possible, albeit labeled by alternative terms of assessment, the terms “formative evaluation,” “feedback,” “AfL,” and “assessment as learning,” were used as the key words in this review.

### Previous reviews of formative assessment on reading achievement

From the literature review, eight major reviews on formative assessment were found in this area (Fuchs and Fuchs, 1986; Kluger and DeNisi, 1996; Black and Wiliam, 1998; Hattie, 2009; Kingston and Nash, 2011; Heitink et al., 2016; Klute et al., 2017; Sanchez et al., 2017). However, only four out of the eight comprehensive reviews encompassed the effect sizes of formative assessment in reading achievement (Fuchs and Fuchs, 1986; Black and Wiliam, 1998; Kingston and Nash, 2011; Klute et al., 2017). These four reviews indicated the positive effects of formative assessment on reading achievement, with effect sizes that ranged widely, from +0.22 to +0.7 (Table 1).

**TABLE 1:** Summary of major meta-analysis on effects of formative assessment on reading achievement.

Authors	Years covered	Types of publication	Subjects covered	Grades	Number of studies (reading)	Effect size
Fuchs and Fuchs	1971–1984	Journal article	Reading and a variety of subjects	Elementary, middle/high	13	+ 0.7 (for all subjects)
Black and Wiliam	Unspecified-1998	Journal article	Reading and a variety of subjects	5 years old to university undergraduates	Unspecified	+ 0.4–0.7 (for all subjects)
Kingston and Nash	1988–2011	Journal article	Reading and a variety of subjects	elementary, middle/high	12	+0.32
Klute et al.	1988–2014	Report	Reading and a variety of subjects	Elementary	9	+0.22

Fuchs and Fuchs’ (1986) generated 96 effect sizes from 21 controlled studies, with an average weighted effect size of +0.70. The authors described that 8 of the 21 investigations focused solely on reading, 4 on reading and math, and 1 on reading, math and spelling, with no specific effect size calculated for reading. This meta-analysis focused upon special education as 83% of the 3,835 investigated subjects belonged to the special educational needs (SEN) population. It is inappropriate to generalize the findings to population of students at large. Secondly, 96 effect sizes generated from the 21 controlled studies were derived from analyses of divergent quality as the authors acknowledged. 69 effect sizes were of fair quality and 8 of poor quality which accounted for around 80% of all the effect sizes. Thus, the average effect size of 0.70 from the 21 studies examined was from research that was methodologically unsound (Dunn and Mulvenon, 2009). The limitation of specialized sample groups and the quality of the studies reviewed cast doubts on the validity of the large effect size.

Black and Wiliam’s (1998) review of more than 250 articles related to formative assessment was a seminal piece to prove the positive effects of formative assessment on student achievement. The authors presented eight articles to support their conclusions pertaining to the efficacy of formative assessment without performing any quantitative meta-analysis techniques. The effect size that ranged from 0.40 to 0.70 concluded from their analysis was equivocal and inadequate to be applied in different contexts (Dunn and Mulvenon, 2009; Kingston and Nash, 2011). This review did not clarify the subject-based effect sizes. Hence, no substantiated effect sizes on reading achievement could be retrieved. Nevertheless, this ‘configurative review’ (Sandelowski et al., 2012) did encourage more widespread empirical research in the area of formative assessment (Black and Wiliam, 2018).

Kington and Nash (2011) screened out 13 of over 300 studies in grades K-12 to reexamine the effects between 0.40 and 0.70. Their moderator analyses indicated that the effect size of formative assessment in English language arts (ES = + 0.32) was larger than those in mathematics (ES = + 0.17) and science (ES = + 0.09). Briggs et al. (2012) commented that one of the marked flaws that threatened Kingston and Nash’s conclusion was their study retrieval and selection approach. It might explain the paucity of Kingston and Nash’s research base (Kingston and Nash, 2012). This problem could be solved by referring to some subset of the studies suggested by Black and Wiliam (1998).

The latest meta-analysis involving reading achievement was conducted by the US Department of Education (Klute et al., 2017). The research team identified 23 rigorous studies on reading, math and writing in elementary level to demonstrate the positive effects of formative assessment interventions on student outcomes from 1988 to 2014. Though it was stated that the review identified studies published between 1988 and 2014, their finalized list for reading only updated to 2007. Of the 23 studies in various subject areas, nine focused on reading with an average effect size of +0.22. Interestingly, their report revealed that other-directed formative assessment was more effective (ES = + 0.41) than student-directed formative assessment (ES = –0.15). Other-directed formative assessment encompassed educators or computer software programs, whilst, student-directed formative assessment referred to self-assessment, self-regulation and peer assessment. The novice categories of formative assessment provided new insights into the moderator analyses. This report was rigorous with stringent controls on selection criteria. However, it solely covered the elementary level and restricted the geographical research location in Anglophone countries.

### Moderator variables

To warrant the quality of a meta-analysis, a rationale for the coding schema should be provided (Pigott and Polanin, 2019). Three factors suggested by Cheung et al. (2021) were set to frame the features of formative assessment.

### Methodological factors

Methodological factors describe research design and sample size. One possible factor that might cause variance is the research design of divergent studies (Abrami and Bernard, 2006). Two groups of research designs were identified in this review: RCT (Randomized Control Trial) and QED (Quasi-experimental design). Particularly of concern is that cluster (school-level, classroom-level, and teacher-level) randomized control trial with student-level outcome measure would be coded as quasi-experimental studies. Another potential source of variation may lie in the sample size which was reported to be negatively correlated with effect sizes in studies of reading program (Slavin and Smith, 2009). Following the tradition in a previous meta-analysis (Cheung and Slavin, 2016), this review coded studies with 250 students or less as small sample, the others were taken as large sample.

### Substantive factors

Substantive factors depict the background of a study such as population, context and duration. Six program features identified from some seminal meta-analyses on reading and formative assessment (Klute et al., 2017) were included in this review.

### Student characteristics, grade level and program duration

Students in the included studies were categorized into at-risk or mainstream students. At-risk students referred to students who had reading difficulties or of low performance in common classrooms, others were coded as mainstream students. Grade level was divided into kindergarten, elementary and middle/high levels. Program duration set 1 year as a threshold to classify long and short programs. Programs that lasted for less than 1 year were coded as short; the rest were long.

### Differentiated instruction

Formative assessment is a “gap minder” (Roskos and Neuman, 2012) enabling teachers and students to identify the gap between where students are and where they need to go in their reading development (Wiliam and Thompson, 2007). Consequently, teachers can stay alert to these gaps and differentiate their instruction to various students. Differentiated instruction is taken as an optional component in the formative assessment practice. In our review, there were several teacher’s practices that were coded as “without interventions.” For instance, teachers who kept track of students’ learning gaps without changing their teaching plan, or who just monitored the interim/benchmark assessment results without further action on differentiating or individualizing their teaching to different students aligned with the data from assessment.

### Type of intervention

Tethered to main sources of formative assessment practices (Andrade et al., 2019) in two latest integrated formative assessment meta-analyses (Klute et al., 2017; Lee et al., 2020), type of intervention was coded as teacher-directed, student-directed or integrated (teacher and student assessment). Specifically, teacher-directed assessment referred to teachers who provided feedback, interim/benchmark assessment or other resources to gauge students’ learning, be it computer-based or paper-based, and/or conducted individualizing or differentiating instruction to students’ classroom learning. Student-directed assessment mainly manifested in the forms of peer- or self- assessment, and young learners’ meaning-focused group reading activity (Connor et al., 2009). Integrated practices involved both teacher and student in the assessment process.

### Digital technology

Various digital technologies have been explored and applied in K-12 formative assessment practice in the 21st century (Spector et al., 2016). A newly published article suggested digital technology could be conducive to reading for young children not but for older children (See et al., 2021). This review will cross check this result by including more rigorous studies on reading from various cultural contexts.

### Other factors

Other factors are the external variables that might influence the variance of effect sizes. We included publication type and cultural settings which were never assessed in previous meta-analyses on formative assessment.

### Publication type

Validity of the results from a meta-analysis is often reported to be threatened by the presence of publication bias. To put it succinctly, publication bias refers to studies with large or statistically significant effects compared to studies with small or null effects being prone to publication. This meta-analysis included both published and unpublished literature (technical reports, dissertations and conference reports).

### Cultural settings

Formative assessment was introduced and developed in Anglophone culture represented by United Kingdom and United States. In light of previous reviewed policies in Asia-Pacific regions, it is safe to assume that formative assessment has been introduced and implemented in Asia, especially in countries or regions heavily influenced by Confucian-heritage culture (CHC) which was heavily influenced by exam-orientation (Biggs, 1998). Teachers from CHC culture are often burdened with high-stake test pressure. It might be more demanding for teachers in CHC classrooms to believe that formative assessment is to facilitate learning rather than accredit it (Crossouard and Pryor, 2012). This review, as a first of its kind, attempted to compare the interventions in Anglophone and Confucian-heritage culture. Studies conducted in Anglophone culture are from Barbados (1), Germany (3), Spain (1), Sweden (1), United Kingdom (1) and United States (30), while studies in CHC settings are from Hong Kong (4), South Korea (1) and Taiwan (3). To note, although Germany, Spain and Sweden are not English-speaking countries, we still categorized them into Anglophone culture in stark contrast to the exam-driven CHC. Surprisingly, few studies from Mainland China could be located to meet our inclusion criteria. The reasons for this were threefold. First, some marginally qualified studies were carried out by only one teacher in two classes so the teacher effect could not be evened out. Second, some studies did not report results of reading achievement as they were not statistically significant, which was explicitly stated by the authors. Besides, the majority of formative assessment projects in China were based in higher education. The culling process implied some new directions for future research and reviews.

Methodological and other factors are mainly the extrinsic factors that can be applied to meta-analyses in other research fields. The substantive factors include intrinsic features that are commonly seen in formative assessment activities. These moderators provide a comparatively holistic set of features that might influence the effect of formative assessment on students’ reading achievement.

### Rationale for present review

Due to the paucity of studies, lack of stringent selection criteria and limitation of samples, the aforementioned comprehensive reviews encouraged more rigorous studies to be investigated to reveal the latest effect size for the subject–reading. The existing subject-based reviews have covered mathematics (Gersten et al., 2009; Burns et al., 2010; Wang et al., 2016; Kingston and Broaddus, 2017), writing (Graham et al., 2015; Miller et al., 2018), and science (Hartmeyer et al., 2018), but not reading.

To provide a more comprehensive understanding of the effectiveness of formative assessment for enhancing reading achievement, this study attempted to elicit exemplary formative assessment practices by applying rigorous, consistent inclusion criteria to identify high-quality studies. Our review, in an effort to sketch a comprehensive picture of the effects of formative assessment on reading, statistically consolidated the effect sizes of qualified studies in terms of methodological and substantive features. The present study attempts to address two research questions:

(1) What is the effect size of formative assessment on K-12 reading programs?
(2) What study and research features moderate the effects of formative assessment interventions on student reading achievement?

## Method

The present review employed meta-analytic techniques suggested by Glass et al. (1981) and Lipsey and Wilson (2001). Comprehensive Meta-analysis Software Version 3.0 (Borenstein et al., 2013) was adopted to compute effect sizes and to carry out various meta-analytical tests. The following steps were taken during meta-analytic procedures: (1) scan potential studies for inclusion using preset criteria; (2) Locate all possible studies; (3) code all qualified studies based on their methodological and substantive features; (4) calculate effect sizes for all selected studies for additional combined analyses; (5) perform comprehensive statistical analyses encompassing both average effects and the relationships between effects and study features.

### Criteria for inclusion

To be included in this review, the following inclusion criteria were preset.

(1) Studies that examined the effects of formative assessment or AfL on students’ reading outcomes.
(2) Studies can be directed by a single party, be it teacher or student (peer- or self- assessment), or by collaboration of teachers and students.
(3) Classroom practices align with the definition of formative assessment in this review.
(4) The studies involved students in kindergarten, elementary and secondary education.
(5) Reading programs included English as a native or a foreign language in their reading courses, or reading courses in students’ mother tongue.
(6) Studies could have taken place in any country or region, but the report had to be available in English or Chinese.
(7) Treatment/experiment group(s) embedded with formative assessment activities was/were compared with control group(s) using standard/traditional methods (aka business-as-usual groups).
(8) Pretest data had to be provided (What Works Clearinghouse, 2020), unless studies used random assignment of at least 30 units (individuals, classes, or schools) and no indications of initial inequality were reported, which were set aligned with ESSA (Every Student Succeeds Act) evidence standards (ESSA, 2015). Studies with pretest differences of more than 50% of a standard deviation were excluded because, large pretest differences could not be adequately managed as underlying distributions may be fundamentally different even with analyses of covariance (Shadish et al., 2002).
(9) Two teachers (each in one classroom) should be involved in each treatment group to even out the teacher effect in treatment effects. Of note, some studies which only examined the students’ roles in formative assessment with only one teacher in each group were included.
(10) Studies interventions had to be replicable in realistic school settings (i.e., in usual classroom setting, students with their usual teacher, controlled experiments). Studies equipping experimental groups with extraordinary amounts of aids (e.g., additional staff to ensure proper implementation) where the Hawthorn effect would be generated were excluded.

### Literature search procedures

All qualified studies from the current review come from three main sources. (1) Previous reviews; Analyzed studies from the previous reviews were further examined. (2) Electronic searches; A comprehensive literature search of articles written up to 2021 was conducted to screen out qualifying studies. Electronic searches were carried out through educational databases (e.g., ERIC, EBSCO, JSTOR, Psych INFO, ScienceDirect, Scopus, Dissertation Abstracts, ProQuest, WorldCat, CNKI), web-based repositories (e.g., Google, Google Scholar), and gray literature databases (e.g., OpenGrey, OpenDOAR). The key words for the search included ‘formative assessment,’ ‘formative evaluation,’ ‘feedback,’ ‘assessment for learning,’ ‘assessment as learning,’ ‘curriculum-based assessment,’ ‘differentiated instruction,’ ‘portfolio assessment,’ ‘performance assessment,’ ‘process assessment,’ ‘progress monitoring,’ ‘response to intervention’ (Gersten et al., 2020), as well as the subset forms under the formative assessment umbrella suggested by Klute et al. (2017) (e.g., self-monitoring, self-assessment, self-direct, peer assessment). (3) Relevant contextualized assessments. The following contextualized assessment projects and systems were included in the searching procedure: learning-oriented assessment (Carless, 2007), A2i (Assessment to instruction) (Connor et al., 2007), SLOA (Self-directed Learning Oriented Assessment) (Mok, 2012), LPA (learning progress assessment) (Förster and Souvignier, 2014), DIALANG (Diagnostic Language Assessment) (Zhang and Thompson, 2004) and CoDiAs (Cognitive Diagnostic Assessment System) (Leighton and Gierl, 2007).

**FIGURE 1:**
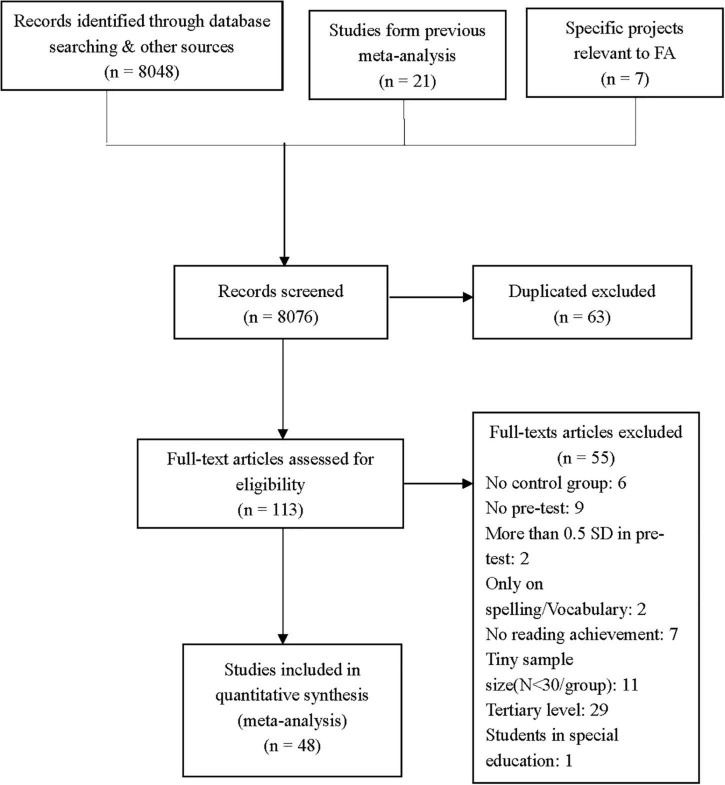
PRIMA flow chart (Moher et al., 2009).

Articles found in the databases were primarily screened by the lead author at the title and abstract level if the purpose of the study matched the independent (formative assessment intervention program) and dependent (reading outcome) variables guiding this meta-analysis. Records identified through database searching numbered 8,048. Additionally, 21 studies were found from previous meta-analysis (Kingston and Nash, 2011; Klute et al., 2017) and a literature review (Lane et al., 2019). Seven studies were included from two formative assessment projects: A2i (Assessment to instruction) (Connor et al., 2007, 2011, 2013; Al Otaiba et al., 2011) and LPA (learning progress assessment) (Förster and Souvignier, 2014, 2015; Förster et al., 2018; Peters et al., 2021). The screening of titles resulted in the retention of 8076 articles at the title and abstract levels that were further examined for eligibility and inclusion in this study. In the first round of screening, we mainly parsed out studies that were not experiments and irrelevant to reading. Then, 113 articles were retained for full-text examination. By applying the inclusion criteria in this review, full-text articles were excluded for the following reasons: without a control group (e.g., Topping and Fisher, 2003), no pre-test (e.g., Cain, 2015), with over 0.50 SD in pre-test (e.g., Hall et al., 2014), only focusing on spelling or vocabulary (e.g., Faber and Visscher, 2018), without reading achievement (e.g., Marcotte and Hintze, 2009), with sample size less than 30 participants (e.g., Chen et al., 2021), students in special education (e.g., Fuchs et al., 1992), and at tertiary level (e.g., Palmer and Devitt, 2014). The numbers in each category can be seen in Figure 1.

### Coding scheme

To assess the relationship between effects and studies’ methodological and substantive features, studies were coded. Methodological features referred to research design and sample size. Substantive features entailed types of publication, grade levels, types of intervention, program duration, implementation, cultural settings, year of publication, students’ characteristics, online technology. The study features were categorized as follows:

(1) Students’ characteristics: Mainstream or at-risk students.
(2) Grade levels: Kindergarten, Elementary (Grade 1–6), Middle/High (7–12).
(3) Types of intervention: teacher-directed (feedback to teacher, response to intervention), student-directed (peer- or self- assessment), integration of teacher and student assessment.
(4) Digital technology: with or without.
(5) Program duration: short (less than 1 year), long (≥1 year).
(6) Differentiated instruction: with or without, and not applicable for those studies only involved peer- and self- assessment that did not describe teachers’ instruction adjustment.
(7) Research design: QED (quasi-experimental design) or RCT (randomized control trial).
(8) Sample size: Small (*N* ≤ 250 students) or large (*N* > 250).
(9) Publication type: published or unpublished.
(10) Cultural settings: Anglophone culture (Australia, Canada, Ireland, New Zealand, United Kingdom, and United States), CHC (Mainland China, Hong Kong SAR, Taiwan, Singapore, Japan, Korea).

The coding of all characteristics was processed by two researchers independently. Inter-rater reliability was calculated by selecting 20 percent of randomly selected studies. Reliability was 87.21 percent. Disagreements were discussed and rectified in light of the definition proposed. All features of formative assessment are presented in Table 2 and descriptive data of qualified studies can be found in the Supplementary material.

**TABLE 2:** Coding scheme features.

Categories of features	Features of FA	Variables
Substantive factors	Student characteristic	(1) Mainstream students
		(2) At-risk students
	Grade level	(1) Kindergarten
		(2) Elementary (1–6)
		(3) Middle/High (7–12)
	Type of intervention	(1) Teacher-directed
		(2) Student-directed (self-assessment)
		(3) Integrated
	Digital technology	(1) Yes
		(2) No
	Program duration	(1) Short (<1 year)
		(2) Long (≥1 year)
	Differentiated instruction	(1) Yes
		(2) No (3) Not applicable (student-directed assessment only)
Methodological factors	Research design	(1) RCT (randomized controlled trial)
		(2) QED (quasi-experimental design)
	Sample size	(1) Large (*N* > 250)
		(2) Small (*N* ≤ 250)
Other factors	Publication type	(1) Published (2) Unpublished
	Cultural setting	(1) Anglophone culture
		(2) Confucian-heritage culture (CHC)

### Effect size calculations and statistical analyses

In general, effect sizes were calculated as the difference between experimental and control student posttests after adjusting for pretests and other covariates, divided by the unadjusted posttest pooled standard deviation. When unadjusted pooled standard deviation was not available, as when the only standard deviation presented was already adjusted for covariates or when solely gain score standard deviations were available, procedures proposed by Sedlmeier and Gigerenzer (1989) and Lipsey and Wilson (2001) were used to estimate effect sizes. Provided that pretest and posttest means and standard deviations were presented but adjusted means were not, effect sizes for pretests were subtracted from effect sizes for posttests. An overall average effect size was produced for each study as these outcome measures were not independent. Comprehensive Meta-Analysis software was employed to carry out all statistical analyses, such as Q statistics and overall effect sizes.

## Results

### Overall effects

**FIGURE 2:**
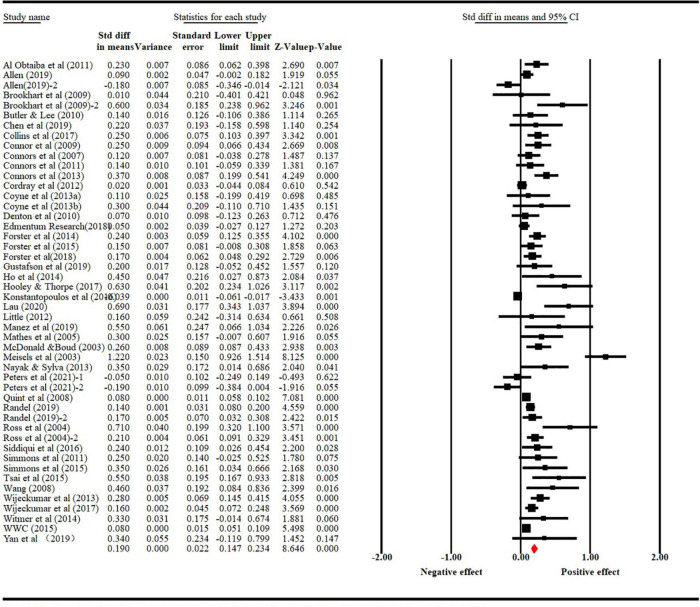
Forest plot of formative assessment effect size on K-12 students’ reading achievement.

A total of 48 qualifying studies was included in the final analysis with a total sample size of 116, 051 K-12 students: 9 kindergarten studies (*N* = 2,040), 28 elementary studies (*N* = 107,919), 11 middle/high studies (*N* = 6,092). The overall effect sizes were calculated in fixed and random effect models. The large *Q* value (*Q* = 313.56, df = 47, *p* < 0.000) indicated that the distribution of effect sizes in this scope of studies is highly heterogeneous. In other words, the variance of study effect seizes is larger than can be explained by simple sampling error. Thus, a random effects model was adopted (DerSimonian and Laird, 1986; Borenstein et al., 2009; Schmidt et al., 2009). As shown in Table 3, the overall weighted effect size is + 0.18 with confident interval between 0.14 and 0.22. In an attempt to interpret this variance, key methodological features (sample size, research design), substantive features (student characteristics, program duration, types of intervention, grade level, digital technology involvement) and extrinsic features (publication type, culture) were used to model some of the variances. An overview of the effect sizes can be seen in Figure 2 that provides a graphical representation of the estimated results of all included studies.

**TABLE 3:** Overall effect size.

	*k*	ES	SE	95% confidence interval		Test of mean		Test of heterogeneity in effect size		
						
				Lower limit	Upper limit	*Z*-value	*p*-value	*Q*-value	df (Q)	*p*-value
(1) Fixed	48	0.07	0.01	0.05	0.08	10.78	0.00	313.56	47	0.000
(2) Random	48	0.19	0.02	0.15	0.23	8.65	0.00			

### Subgroup analysis

The heterogeneity in the overall effect calculation implies that the large differences between these 48 included studies might be related to researchers’ choice of methodology, samples’ substantive features and other factors. Before our meta-regression analysis, we first estimated the comparisons in subgroups as shown in Table 4. Variation yielded significant differences in effect sizes that were from seven moderators: grade level, type of intervention, program duration, differentiated instruction, sample size, publication type and cultural setting. The effect sizes comparisons of subcategories in rest three moderators, student characteristics, digital technology and research design were non-significant.

**TABLE 4:** Subgroup analysis results.

	Features	Studies included (*k*)	Effect size	*p*
Student characteristic	Mainstream students	37	0.18	0.156
	At-risk students	11	0.27	
Grade level*	Kindergarten	9	0.28	0.038
	Elementary (1–6)	28	0.16	
	Middle/High (7–12)	11	0.27	
Type of intervention*	Integrated	19	0.20	0.011
	Teacher-directed	19	0.12	
	Student-directed	10	0.31	
Digital technology*	No	19	0.32	0.011
	Yes	29	0.15	
Duration	Long	25	0.16	0.110
	Short	23	0.21	
Differentiated instruction***	Yes	29	0.24	0.000
	No n.a.	9 10	0.05 0.36	
Research design	QED	36	0.18	0.382
	RCT	12	0.22	
Sample size***	Large	30	0.13	0.000
	Small	18	0.45	
Publication type***	Published	38	0.26	0.000
	Unpublished	10	0.09	
Cultural setting**	Anglophone	40	0.17	0.005
	Confucian-heritage culture	8	0.38	

*p < 0.05, **p < 0.01, ***p < 0.001.

The reason we performed subgroup analysis was to provide basic descriptive data of the ‘constructed’ features in formative assessment. Six substantive features have been investigated in previous reviews (Kingston and Nash, 2011; Klute et al., 2017; Lee et al., 2020). In this review, we added methodological factors (research design and sample size) and other factors (publication types and cultural settings) to examine how effect size of formative assessment on reading would vary in these categories. Hence, after we added new moderators into the meta-regression model, the subgroup analysis data would help us decipher why some of the results were contrary to previous findings.

### Meta-regression

To address the second research question, we regressed all the moderator variables in a model presented in Table 5 to describe the predicted different standard deviation (coefficient) of comparing the categories in each moderator after controlling for other features of formative assessment interventions. It is assumed that the effects from meta-regression are more confidently reliable than results from subgroup analysis by taking account of the iterative influences from different moderators. In our proposed model, only three pairs of moderator categories comparison were significant.

**TABLE 5:** Results of meta-regression.

Random effects	Coefficient	SE	*p*
Intercept	0.07	0.08	0.409
Sample size (Small) ***	0.33	0.06	0.000
Type of intervention (Teacher-directed) ***	–0.12	0.03	0.000
Type of intervention (Student-directed)	0.03	0.09	0.769
Differentiated instruction (Yes) ***	0.13	0.03	0.000
Differentiated instruction (n.a.)	0.17	0.11	0.124
Research design (RCT)	–0.04	0.06	0.465
Digital technology (Yes)	0.001	0.04	0.978
Grade level (Elementary)	0.02	0.07	0.790
Grade level (Middle/High)	–0.03	0.07	0.696
Student characteristics (at-risk students)	–0.001	0.08	0.983
Duration (Short)	0.02	0.04	0.576
Cultural settings (CHC)	–0.10	0.09	0.297
Publication type (unpublished)	0.01	0.03	0.701
*Q*	99.73		0.000
Df *R*^2^ analog	13 0.95		

*p < 0.05, **p < 0.01, ***p < 0.001.

First, the effect size from different *sample sizes* varied substantially. As aforementioned, we set *N* = 250 as a cut-off point. Clearly, small sample size studies yielded a significantly larger effect size (*d* = 0.33, *p* < 0.001) than large sample studies.

Next, three *types of intervention* were examined, namely, teacher-directed, student-directed and integration of teacher and student assessment. Results indicated that, when other features of formative assessment were controlled, formative assessment only engaged by teachers had a significantly smaller effect size than integrating teacher and students’ assessment in the intervention for reading (*d* = –0.12, *p* < 0.001), whereas, student-directed assessment (self- assessment) showed no significant difference (*d* = 0.03, *p* = 0.769).

Third, in regard to *differentiated instruction*, we coded formative assessment in reading with or without differentiated instruction. Some interventions that only involved student directed assessment without teachers’ instructional adjustment were coded as not applicable (n.a.). Results, as we hypothesized, favored teachers who used differentiated instruction during or after their formative assessment on students’ reading. If a teacher adopted differentiated or individualized instruction during or after formative assessment, students’ reading achievement would be significantly higher than those of their peers taught by a teacher only applied formative assessment (*d* = 0.13, *p* < 0.001). When formative assessment was directed by the student, the effect size on reading achievement was larger than formative assessment with teachers’ differentiated instruction, albeit not significantly (*d* = 0.17, *p* = 0.124).

Apart from the three pairs of contrast, the rest of the moderator variables comparisons were non-significant, although some showed significant results in subgroup analysis.

Given that *research design* might influence the effect size, we categorized all studies into randomized controlled studies (RCT) and quasi-experiments (QED). Results from the regression model indicated that effect sizes generated from RCT were smaller than QED design, but not significant (*d* = –0.04, *p* = 0.465).

*Digital technology* involvement was examined. Surprisingly, students’ reading achievement was not influenced significantly by formative assessment with digital technology (*d* = 0.001, *p* = 0.978).

Formative assessment in reading classrooms seemed to exert a similar impact on students of different *grade level*s. The effect sizes in elementary level were slightly larger than those for kindergarten studies (*d* = 0.02, *p* = 0.790). Effect sizes for middle/high school studies were also slightly smaller than those for kindergarten studies (*d* = –0.03, *p* = 0.696).

*Student characteristics* were coded into mainstream students and at-risk students. The estimated effect size of formative assessment on mainstream students was slightly higher than that on at-risk students, albeit not significant (*d* = 0.001, *p* = 0.982).

With respect to *program duration*, studies that lasted less than 1 year were coded as short programs, others were coded as long ones. Programs in one-year or longer showed smaller effect size than short-term ones, but the difference was non-significant (*d* = –0.02, *p* = 0.576).

Different from the result of the subgroup analysis result regarding *cultural setting*, the effect size of formative assessment in CHC appeared to be smaller than those in Anglophone culture, though not significant (*d* = –0.10, *p* = 0.297).

Results of publication type revealed that no significant differences were found between published and unpublished articles (*p* = 0.701), indicating that no publication bias existed in this review.

The pseudo *R*^2^ value in this meta-regression model estimated the moderators accounted for 95% of heterogeneity. The predictive power of this value is reliable as the number of studies (*k*) in this review exceeded the minimum number of 40 as suggested by López-López et al. (2014).

## Discussion

### Overall effect size

The findings of this review indicate that formative assessment produce a positive effect (ES = + 0.18) on reading achievement. The magnitude could be interpreted as a *small* effect aligned with the oft-cited indication of small (*d* = 0.2), medium (*d* = 0.5), and large (*d* = 0.8) effect size (Cohen, 1988). However, Kraft (2020), taking study features, program costs and scalability into account, proposed a new benchmark frame for effect size from causal studies of pre K-12 education intervention, namely small (*d* < 0.05), medium (0.05 to <0.20), and large (≥0.20). Accordingly, the overall aggregated effect size in this review could be taken as a medium effect size. Compared with effect sizes (from +0.22 to +0.70) from previous meta-analyses, the weighted average effect size reported in this review was the smallest one. Two potential factors may explain this. First, some early reviews set comparatively looser criteria for inclusion, which often inflates effect size estimates. Pertaining to our set of stricter inclusion criteria, five studies in Klute et al. (2017) review with less than 30 participants in each group (Fuchs et al., 1989; McCurdy and Shapiro, 1992; Johnson et al., 1997; Iannuccilli, 2003; Martens et al., 2007) were ruled out in the present review. Second, 35 of our selected studies were conducted after 2010 whereas a latest previous meta-analysis (Klute et al., 2017) only included studies till 2007. As publication bias might be mitigated over time (Guan and Vandekerckhove, 2016), more insignificant or even negative findings were reported. In this review, two large scale studies (Konstantopoulos et al., 2016; Allen, 2019) involving over 35,000 student reported negative effects of formative assessment on reading achievement.

### The effects of moderators

The meta-regression results indicate that sample size, differentiated instruction and type of intervention suffice to account for the heterogeneity of the effect sizes. Additionally, we intend to discuss some implications from the results of cultural settings, digital technological and publication bias.

### Sample size

Prior research indicated that studies with small sample sizes tend to yield much larger effect size than do large ones (Liao, 1999; Cheung and Slavin, 2016). In this review, sample size was a crucial variable that might influence the effect size of formative assessment on reading achievement. Two explanations could be put forward for this result. First, intuitively, small-scale studies are more likely able to be implemented with high fidelity. Teachers might find it easier to give more support for students and monitor their progress. Researchers are more likely to purposefully recruit motivated teachers and schools. In this sense, they tend to produce larger effect size than large-scale studies. Next, researchers using small samples would be apt to more design self-developed outcome measures (Wang, 2008; Tsai et al., 2015; Lau, 2020; Yan et al., 2020)., which might be more sensitive to treatments than standardized studies (Cheung and Slavin, 2016).

### Differentiated instruction

One of the key findings in our review was the positive effects of differentiated instruction during or after formative assessment on reading achievement for K-12 students. This significant result is in accord with the findings from an influential U.S. data-driven reform model on state assessment program. Slavin et al. (2013) found that, for fifth-grade reading, those schools and teachers adjusting reading instruction produced educationally important gains in achievement, while others did not if they merely understood students’ data without further action on instructional adjustment. Formative assessment was analogous to taking a patient’s temperature, while differentiated instruction was analogous to providing a treatment (Slavin et al., 2013).

In a study included in our review with a comparatively promising large effect size (*d* = + 0.63) on an early literacy program designed for students at-risk, the researchers concluded that “if one practices formative assessment seriously, one will necessarily end up differentiating instruction” (Brookhart et al., 2010, p. 50). In a recent review on formative assessment (Lee et al., 2020), the research team coded a similar moderator “instructional adjustment” and revealed no significant contrast between their four moderator variables: no adjustment, planned adjustment, unplanned adjustment and mixed. We assumed that the effects might be ameliorated if too many variables were coded which led to the insufficient numbers in each category. Additionally, in our own model, primarily we added professional development as a moderator. However, this moderator was highly correlated with “differentiated instruction.” Meta-regression could not be computed due to the collinearity. It is worth mentioned, in our qualified studies, 94% (34/36) of the interventions embedded with differentiated instruction were coupled with professional development for teachers. The evidence in turn implied that professional development is vital in fostering high fidelity of implementing formative assessment on reading programs.

### Type of intervention

The types of intervention result indicated that an integration of teacher-directed and student-directed would be more effective than formative assessment in reading program directed by teacher or student alone in K-12 settings. In a previous meta-analysis, the research teamed concluded that other-directed formative assessment that encompassed educators or computer software programs was more effective than student-directed formative assessment (Klute et al., 2017). They included nine studies in their review, six of which were designed for students with special education needs. These participants might be less capable of making self- or peer-directed formative assessment. In the present review, a more holistic picture was depicted for general population was obtained advocating an integrated usage of teacher-directed and student-directed assessment.

The results of our review suggested that integrating teacher and student in formative assessment might be more effective than teacher- or student- directed assessment to enhance students’ reading achievement. We attempted to explained this based on linguistic theory (Kintsch and Van Dijk, 1978). Some production-based subject like writing might be more effective when the formative assessment was student-centered (Black and Wiliam, 1998), but reading is a comprehension-based subject that requires explicit instruction necessitated by teachers’ guidance (McLaughlin, 2012). Also, feedback messages require students’ active construction on deciphering with the help of teachers (Ivanic et al., 2000; Higgins et al., 2001). But we were given a caveat that it was not a “one size fits for all” suggestion from our screening on studies in Anglophone and Confucian-heritage cultures.

### Cultural setting

The subgroup analysis comparison result of interventions in two cultures were significant. Studies conducted in Confucian heritage culture yielded ostensibly much larger sample sizes than those in Anglophone culture. Nevertheless, the non-significant data in meta-regression indicated that it was influenced by other variables. By drawing on the data and the evidence we collected, we found it hard not to associate the impact with sample size. All the qualified studies in CHC were of small sample size. Sample size was reported to be one of the significant moderators which contributed to the variance of effect sizes in this review. After controlling for other moderators, no significant differences were found between the interventions in these two cultures.

Though it was provisional to conclude that there was no difference between the studies in Confucian-heritage culture and Anglophone culture, our screening process and descriptive data in subgroup analysis might render us some hints for the interpretation of the results.

Only eight qualified studies were set in CHC, while 38 for Anglophone culture. The limited number of experimental studies from CHC settings might be associated with the barriers of formative assessment intervention in CHC. Teachers from CHC (Mainland China, Hong Kong SAR, Taiwan, Singapore, Japan, Korea) are often challenged by large class sizes (Hu, 2002) and high-stake test pressure (Berry, 2011), which gives rise to teachers’ psychological burden on assessment (Chen et al., 2013). These sociocultural factors drastically hinder the translation (local adaptation of an educational policy) (Steiner-Khamsi, 2014) of formative assessment. When a school advocates formative assessment for teachers without appropriate professional development, they take it as a “villain of workload” (Black, 2015). Teachers in a test-driven culture would inevitably take formative assessment as a “test” instead of instruction.

Next, particularly of concern is the hint we obtained from the promising results of our included studies in CHC. Researchers in CHC contexts have started to explore alternative ways to implement formative assessment. Six out of eight studies in CHC in our review were self-assessment (Wang, 2008; Butler and Lee, 2010; Tsai et al., 2015; Chen et al., 2017; Lau, 2020; Yan et al., 2020). This renders us a new direction that self- assessment might be alternatives for reading teachers to implement formative assessment as part of their teaching in CHC classrooms. But we are far from confident to conclude it’s the most effective way based upon the data we reported in this review.

### Digital technology

Previous meta-analysis findings revealed that mobile devices (Sung et al., 2016) and educational technology (Slavin, 2013) do not exert significant differences on students’ academic achievement, and digitally delivered formative assessment is only conducive to reading for young school-age children but not for older children (See et al., 2021). In line with those reviews, our findings also indicated that formative assessment with digital technology does not significantly influence students’ reading achievement compared with traditional paper-pen intervention. The findings caution that digital technology is not the kernel of formative assessment. Nevertheless, our findings still advocate technology-enhanced formative assessment as it can provide an evidence-based platform to scaffold students’ learning by generating and deploying formative feedback. From the methodological perspective, computer-based formative assessment systems are generally more accessible for teachers and students than traditional methods (Tomasik et al., 2018). Of note, lessons can be drawn from the undesirable effect sizes of those digital formative assessment programs: (1) A digital formative assessment program can be promisingly effective when teachers in intervention group differentiate their instructional practices based on the evidence feedbacked by the digital program. Researchers from the benchmark or interim assessment with small (Cordray et al., 2013) or even negative effect sizes (Konstantopoulos et al., 2016) reflected that teachers might need further support to adjust their teaching as their classroom schedules were quite crowded. (2) Professional development and training for teachers participating in the digital formative assessment are irreplaceable prerequisites for the quality of practice. Support for teachers to understand the concept and provision of technical assistance are essential for their instructional change (Connor et al., 2009; Kennedy, 2009).

### Publication bias

To mitigate the threat of publication bias, we included 10 unpublished studies in this review. Traditional methods to assess publication bias included a visual inspection of symmetric dispersion of a funnel plot (Sterne et al., 2005), “fail safe N” statistics (Orwin, 1983), trim-and fill method (Duval and Tweedie, 2000) and setting publication bias as a moderator to test the differences in mean effect sizes between published and unpublished studies (Polanin and Pigott, 2015). As the latter method was comparatively straightforward and more objective than eyeballing evaluation, we took publication bias as a moderator in meta-regression to compare the mean effect sizes of published and unpublished studies by controlling other factors. No significant difference was found between the two groups of studies. We believe that publication bias is not a concern for the current meta-analysis.

## Conclusion

This review has revealed that, without publication bias, formative assessment is making a positive and modest difference in enhancing students’ reading achievement in diverse settings. The average weighted effect over all included studies was 0.19. The exact size a researcher finds may deviate considerably depending on the sample size, teachers’ differentiated instruction and type of intervention. Studies involving a large sample size with over 250 students led to low and attenuated estimate of formative assessment. The implementation of teachers’ differentiated instruction is linked to much stronger effects than intervention without differentiated instruction. Also, our results suggested that collaboration of teachers and students in formative assessment would be more effective than formative assessment merely initiated by teachers. Findings suggest that teachers are strongly encouraged to adjust their reading instruction in terms of content, process and product catering to student diversity (Tomlinson, 2001) during the formative assessment in the cooperation with students themselves. Studies with differentiated instruction coupled with teacher’s professional development has a positive and modest effect on reading outcome. To enhance students’ reading achievement and upskill teachers, future studies designs should focus more on effective components that facilitate differentiated instruction and professional development.

This meta-analysis contributes to the existing understanding about formative assessment in K-12 reading program in three significant ways. First, it systematically records the critical components of formative assessment pertaining to reading program for frontline teachers to refer to by catering for learner diversity (Snow, 1986). Second, it affords a new cross-cultural perspective by comparing western and eastern formative assessment practices for school administrator and policy makers to tailor effective programs in their unique cultural contexts. Lastly, it substantiates the discipline-specific characteristics in reading to conceptualize formative assessment for K-12 reading program (Bennett, 2011), which is pivotal to a next-generation definition of domain-dependent formative assessment (Cizek et al., 2019).

It is vital to mention several limitations of this review merely focusing on the quantitative measurement of reading achievement. Evidence-based education advocates the insightful and irreplaceable findings from qualitative research (Slavin and Cheung, 2019). There is much to learn from non-experimental studies that can interpret the effects of formative assessment on students’ reading. Next, this review centered on a standardized test of reading achievement. However, other outcomes maybe of great value to policymakers and practitioners. Third, student-directed assessment is often referred to peer- or self-assessment. Third, the qualified studies in this meta-analysis only include self-assessment. We are aware the value of peer-assessment and strongly suggest future review could locate more qualified studies concerning this type of assessment. Lastly, the culture settings in this study merely include Anglophone or CHC as we could not locate acceptable studies from other cultures temporarily. Studies setting at all cultures were equally important and should be included if possible. Further studies could explore research from other cultures.

Educational borrowing from other countries is not a simple case of duplicating the successful tales, inasmuch as extrapolation and recontextualization of educational interventions are embedded with cultural and historical stories (Luke et al., 2013). Our subgroup analysis indicated cultural settings might be a potential moderator. As a wealth of large-scale formative assessment initiatives have been advanced in classrooms heavily influenced by CHC, synthesized effect sizes in CHC settings are encouraged to be reported to ensure the continuity of formative assessment with cultural script (Stigler and Hiebert, 1998, 2009). Future reviews can apply narrative synthesis methods to explore the factors that advance or hinder the development of formative assessment on reading in CHC. Considering the complicated implementation of formative assessment on reading (Lane et al., 2019), teachers in CHC classrooms are suggested to explore their own ways to effectively “import” (Xu and Harfitt, 2018) and “translate” high-quality formative assessment (Black and Wiliam, 1998).

## Author contributions

QX conceived of the presented idea. QX and AC performed the analytic calculations, performed the numerical simulations and contributed to the final version of the manuscript. DS contributed to important intellectual content in the revised version and the final version of the manuscript.
