# Supplementary material for: The effectiveness of formative assessment for enhancing reading achievement in K-12 classrooms: A meta-analysis
Source: Front Psychol. 2022 Aug 22;13:990196. doi: 10.3389/fpsyg.2022.990196 (PMC9443994; doi:10.3389/fpsyg.2022.990196)
Supplement: Supplementary file 1 [file Table_1.DOCX]

**Appendix A**

**19 Definitions of formative assessment in the literature**

|  | What | Why | When | Who | How |
| --- | --- | --- | --- | --- | --- |
| Sadler, 1989, p.120 | judgements about the quality of student responses (performances, pieces, or works) | to shape and improve the students’ competence |  |  | short-circuit the randomness and inefficiency of trial-and-error learning |
| Black & Wiliam, 1998, p.7-8 | activities | to adapt the teaching to meet students’ needs |  | teachers and students | to assess themselves by activities that provide information to be used as feedback to modify teaching and learning activities |
| Cowie & Bell, 1999, p.32 | a process | to enhance that learning | during the learning | teachers and students | to recognize and respond to student learning |
| Broadfoot et al. 2002, p 2-3 | a process | to decide where the learners are in their learning, where they need to go and how best to get there |  | teachers and learners | to seek and interpret evidence for use by learners and their teachers |
| Kahl, 2005, p.11 | a ‘midstream’ tool |  | while the material is being taught | teachers | to measure student grasp of specific topics and skills they are teaching and to identify specific student misconceptions and mistakes |
| Looney, 2005, p.21 | frequent, interactive assessments of students’ progress and understanding |  |  |  | to identify learning needs and adjust teaching appropriately |
| Shepard et al, 2005, p.275 | assessments | to improve teaching and learning | during the instructional process |  |  |
| Stiggins, 2005, p.4 | A set of different assessment methods (assessment for learning) |  |  | Teachers, students and parents | To provide students, teachers and parents with a continuing stream of evidence of student progress in mastering the knowledge and skills that underpin or lead up to state standards |
| Popham, 2008, p.6 | a plan process |  |  | teachers and students | to use assessment-elicited evidence of students’ status in which teachers to adjust their ongoing instructional procedures;  students to adjust their current learning tactics; |
| Wiliam & Thompson, 2008, p.15 |  | to identify where learners are in their learning, where they are going and how to get there |  | teachers, peers and learners | to engineer effective classroom discussions, activities and tasks that elicit evidence of learning, to activate learners as instructional resources for one another and the owners of their own learning, and to provide feedback that moves learners forward |
| Black & Wiliam, 2009, p.6 | classroom practices | to make decisions about the next steps in instruction that are likely to be better, or better founded than the decisions they would have taken in the absence of the evidence that was elicited |  | teachers, learners and their peers | to elicit, interpret and use evidence about student achievement |
| Chappius, 2009, p.12 | the formal and informal processes | to adjust teaching learning that merits the formative label |  | teachers and students | to gather evidence for the purpose of improving learning |
| Moss & Brookhart, 2009, p.6 | an active and intentional learning process | to improve student achievement |  | teachers and students | continuously and systematically gather evidence of learning |
| Greenstein, 2010, p.21 | a particular assessment | for further progress towards learning goals |  |  | to track learning, give students feedback and adjust instructional strategies |
| Heritage, 2010, p.8 | a process | to keep learning moving forward to meet the learning goals | during teaching and learning | teachers and students | to gather information to take steps to keep learning moving forward |
| Marzano, 2011, p.8 | a process | to tie to learning goals | while instruction is occurring |  |  |
| Cizek, 2010, p.6 | the collaborative processes | to understand students’ learning and conceptual organization, identification of strengths, diagnosis of weaknesses, areas for improvement, and as a source of information that teachers can use in instructional planning and students can use in deepening their understandings and improving their achievement |  | educators and students |  |
| Popham, 2011, p.2 | a planned process | to improve what they’re doing |  | teachers and students | to use assessment-elicited evidence |
| Cizek et al, 2019, p.14-15 | a source of information as one part of a planned assessment ecosystem; | to support teachers’ and students’ inferences and strengths, weaknesses, and opportunities for improvement in learning; | in educators’ instructional planning and students’ deepening their understandings, improving their achievement, taking responsibility for and self-regulating their learning | educators and students | to include general principles, and discipline-specific elements that comprise formal and informal materials, collaborative processes, ways of knowing, and habits of mind particular to a content domain |

Note. In the table, definitions are listed chronologically.

**Appendix B**

**Descriptive data of 48 included studies**

| Study | FA Tool | Research Design (Sample size) | Type of intervention  (with digital technology) | Differentiated instruction | Country/ region | Sample characteristic (Grade level) | Duration  (unpublished report) | ES |
| --- | --- | --- | --- | --- | --- | --- | --- | --- |
| Al Otaiba et al. (2011) | A2i (Assessment to instruction) | cluster RCT(L) | teacher-directed （Y） | Y | USA | at-risk (K) | 9 months | 0.23 |
| Allen (2019)-1 | ACT Aspire Periodic Assessments | QED(L) | integrated（Y） | N | USA | mainstream (E) | 1 year (U) | 0.09 |
| Allen (2019)-2 | ACT Aspire Periodic Assessments | QED(L) | integrated(Y) | N | USA | mainstream(M/H) | 1 year (U) | -0.18 |
| Brookhart et al. (2010)-1 | Teaching as Intentional Learning | QED(S) | teacher-directed(N) | Y | USA | mainstream(K) | 9 months | 0.01 |
| Brookhart et al. (2010)-2 | Teaching as Intentional Learning | QED(S) | teacher-directed(N) | Y | USA | mainstream(E) | 9 months | 0.63 |
| Butler and Lee (2010) | self-assessment | QED (L) | student-directed(N) | n.a. | South Korea | mainstream(E) | 1 semester | 0.14 |
| Chen et al. (2017) | attention-based diagnosing and review mechanism (ADRM) | RCT(S) | student-directed(Y) | n.a. | Taiwan | mainstream(M/H) |  | 0.22 |
| Collins et al. (2017) | WIRC (Writing Intensive Reading Comprehension) interactive think sheets | cluster RCT(L) | integrated(N) | Y | USA | mainstream(E) | 1 year | 0.46 |
| Connor et al. (2009) | A2i (Assessment to instruction) | cluster RCT(L) | integrated(Y) | Y | USA | mainstream(E) | 1 Year | 0.25 |
| Connor et al. (2011) | A2i (Assessment to instruction) | cluster RCT(L) | integrated(Y) | Y | USA | mainstream(E) | 1 Year | 0.14 |
| Connor et al. (2013) | A2i (Assessment to instruction) | cluster RCT(L) | integrated(Y) | Y | USA | mainstream(K) | 3 years | 0.37 |
| Connor et al. (2007) | A2i (Assessment to instruction) | cluster RCT(L) | integrated(Y) | Y | USA | mainstream(E) | 1 year | 0.12 |
| Cordray et al. (2013) | MAP (Measures of Academic Progress) benchmark testing system | cluster RCT(L) | teacher-directed(Y) | Y | USA | mainstream(E) | 2 years (U) | 0.02 |
| Coyne et al. (2013a) | ERI (Early Reading Intervention) | cluster RCT(L) | teacher-directed(N) | Y | USA | mainstream(K) | 20 weeks | 0.11 |
| Coyne et al. (2013b) | Adjusting ERI (Early Reading Intervention) | cluster RCT(L) | teacher-directed(N) | Y | USA | mainstream(K) | 25 weeks | 0.30 |
| Denton et al. (2010) | RRI (Responsive Reading Instruction) | cluster RCT (S) | teacher-directed(N) | Y | USA | mainstream(E) | 2 years | 0.07 |
| Edmentum (2018) | Study Island | cluster RCT (S) | integrated(Y) | N | USA | mainstream(E) | 1 year (U) | 0.05 |
| Förster and Souvignier (2014) | LPA-G (Learning progress assessment)-Goal setting | QED(L) | integrated(Y) | Y | Germany | mainstream(E) | 6 months | 0.24 |
| Förster and Souvignier (2015) | Learning Progress Assessment (with teaching training) | RCT(L) | teacher-directed(Y) | Y | Germany | mainstream(E) | 6 months | 0.15 |
| Förster et al. (2018) | LPA (Learning progress assessment)-RS (Reading sportsman) | QED(L) | integrated(Y) | Y | Germany | mainstream(E) | 2 years | 0.17 |
| Gustafson et al. (2019) | LegiLexi | QED(L) | teacher-directed(Y) | Y | Sweden | mainstream(E) | 1 year | 0.20 |
| Ho et al. (2014) | Tiered intervention | RCT(S) | teacher-directed(N) | Y | Hong Kong | at-risk(E) | 1 year | 0.45 |
| Hooley and Thorpe (2017) | Curriculum-based reading assessment platform | cluster RCT (L) | student-directed(Y) | n.a. | USA | mainstream(M/H) | 6 weeks | 0.63 |
| Konstantopoulos et al. (2016) | Diagnostic Assessment Tools (DAT)-mClass+ Acuity | RCT(S) | teacher-directed(Y) | N | USA | mainstream(E) | 1 year | -0.04 |
| Lau (2020) | activity worksheet + peer assessment | QED(S) | student-directed(N) | n.a. | Hong Kong | mainstream(M/H) | 1 year | 0.69 |
| Little et al. (2012) | ERI (Early Reading Intervention) | cluster RCT (L) | teacher-directed(N) | Y | USA | at-risk(K) | 20 weeks | 0.16 |
| Máñez et al. (2019) | elaborated feedback (EF) | QED(S) | student-directed(Y) | n.a. | Spain | mainstream(M/H) |  | 0.55 |
| Mathes et al. (2005) | Responsive Reading | cluster RCT (S) | teacher-directed(N) | Y | USA | at-risk(E) | 2 years | 0.30 |
| McDonald and Boud (2010) | self-assessment | RCT(S) | student-directed(N) | n.a. | Barbados | mainstream(M/H) | 1 year | 0.26 |
| Meisels et al (2003) | Work Sampling System (WSS) | QED(S) | Integrated (N) | Y | USA | Mainstream (E) | 1 year | 1.22 |
| Nayak and Sylva (2013) | Guided Reading | RCT(S) | teacher-directed(N) | Y | HK | mainstream(E) | 8 weeks | 0.35 |
| Peters et al (2021) | Learning Progress Assessment | QED(L) | Teacher-directed(Y) | N | Germany | Mainstream(E) | 1 year | -0.05 |
| Peters et al (2021)-2 | Learning Progress Assessment + Reading Sportsman | QED(L) | Teacher-directed(Y) | Y | Germany | Mainstream(E) | 1 year | -0.19 |
| Quint et al. (2008) | FAST-R (Formative Assessments in Student Thinking in Reading) | cluster RCT (L) | teacher-directed(N) | N | USA | mainstream(E) | 1 Year (U) | 0.08 |
| Randel (2019)-1 | Study Island | RCT(S) | Integrated(Y) | N | USA | mainstream(E) | 1 semester (U) | 0.14 |
| Randel (2019)-2 | Study Island | QED(L) | Integrated(Y) | N | USA | mainstream(M/H) | 1 semester (U) | 0.17 |
| Ross et al. (2004)-1 | Accelerated Reader (AR)/Reading Renaissance (RR) | QED(L) | teacher-directed(Y) | Y | USA | mainstream(K) | 1 year (U) | 0. 71 |
| Ross et al. (2004)-2 | Accelerated Reader (AR)/Reading Renaissance (RR) | QED(L) | teacher-directed(Y) | Y | USA | mainstream(E) | 1 year (U) | 0.21 |
| Siddiqui et al. (2016) | Accelerated Reader (AR) | cluster RCT (S) | teacher-directed(Y) | n.a. | UK | mainstream(M/H) | 20 weeks | 0.24 |
| Simmons et al. (2011) | ERI (Early Reading Intervention) | cluster RCT (L) | teacher-directed(N) | Y | USA | at-risk(K) | 20 weeks | 0.25 |
| Simmons et al. (2015) | ERI (Early Reading Intervention) with ongoing adjustments (ERI-A) | RCT(L) | teacher-directed(N) | Y | USA | at-risk(K) | 20 weeks | 0.35 |
| Tsai et al. (2015) | immediate elaborated feedback (IEF) | cluster RCT (S) | student-directed(Y) | n.a. | Taiwan | mainstream(M/H) | 4 weeks | 0.55 |
|  | GAM-WATA (multiple-choice Web-based quiz-game-like formative assessment system) | cluster RCT (S) | student-directed(Y) | n.a. | Taiwan | mainstream(E) | 2 weeks | 0.46 |
| Wijekumar et al. (2013) | ITSS (Intelligent Tutoring System for the Text Structure Strategy) | QED(S) | integrated(Y) | Y | USA | mainstream(E) | 2 semesters | 0.28 |
| Wijekumar et al. (2017) | ITSS (Intelligent Tutoring System for the Text Structure Strategy) | RCT(S) | integrated(Y) | Y | USA | mainstream(M/H) | 22 weeks | 0.16 |
| Witmer et al. (2014) | COCA (the Concepts of Comprehensive Assessment) | cluster RCT (L) | teacher-directed(N) | Y | USA | mainstream(E) | 9 months | 0.33 |
| WWC (2015) | Diagnostic Assessment Tools (DAT) -- mClass+ Acuity | cluster RCT (L) | teacher-directed(Y) | N | USA | mainstream(E) | 1 year (U) | 0.08 |
| Yan et al. (2020) | student diary as self-assessment tool | QED(S) | student-directed(N) | n.a. | HK | mainstream(M/H) | 5 weeks | 0.34 |

Note. In the table, studies are listed alphabetically by authors’ family names. In sample size column, S indicates a small sample (N ≤ 250 participants), L indicates a large sample size (L>250 participants). In Grade level column, K refers to kindergarten, E for elementary school, M/H for middle/high school. Some articles or reports included two studies for different target students were reported with two effect sizes.
